# Multifunctional nanoemulsions for intraductal delivery as a new platform for local treatment of breast cancer

**DOI:** 10.1080/10717544.2018.1440665

**Published:** 2018-03-01

**Authors:** Amanda Migotto, Vanessa F. M. Carvalho, Giovanna C. Salata, Fernanda W. M. da Silva, Chao Yun Irene Yan, Kelly Ishida, Leticia V. Costa-Lotufo, Alexandre A. Steiner, Luciana B. Lopes

**Affiliations:** a Department of Pharmacology, Institute of Biomedical Sciences, University of Sao Paulo, Sao Paulo, Brazil;; b Department of Microbiology, Institute of Biomedical Sciences, University of Sao Paulo, Sao Paulo, Brazil;; c Department of Cell and Developmental Biology, Institute of Biomedical Sciences, University of Sao Paulo, Sao Paulo, Brazil;; d Department of Immunology, Institute of Biomedical Sciences, University of Sao Paulo, Sao Paulo, Brazil

**Keywords:** Intraductal administration, C6 ceramide, nanoemulsion, nanocarrier, breast cancer

## Abstract

Considering that breast cancer usually begins in the lining of the ducts, local drug administration into the ducts could target cancers and pre-tumor lesions locally while reducing systemic adverse effects. In this study, a cationic bioadhesive nanoemulsion was developed for intraductal administration of C6 ceramide, a sphingolipid that mediates apoptotic and non-apoptotic cell death. Bioadhesive properties were obtained by surface modification with chitosan. The optimized nanoemulsion displayed size of 46.3 nm and positive charge, properties that were not affected by ceramide encapsulation (0.4%, w/w). C6 ceramide concentration necessary to reduce MCF-7 cells viability to 50% (EC_50_) decreased by 4.5-fold with its nanoencapsulation compared to its solution; a further decrease (2.6-fold) was observed when tributyrin (a pro-drug of butyric acid) was part of the oil phase of the nanocarrier, a phenomenon attributed to synergism. The unloaded nanocarrier was considered safe, as indicated by a score <0.1 in HET-CAM models, by the high survival rates of *Galleria mellonella* larvae exposed to concentrations ≤500 mg/mL, and absence of histological changes when intraductally administered in rats. Intraductal administration of the nanoemulsion prolonged drug localization for more than 120 h in the mammary tissue compared to its solution. These results support the advantage of the optimized nanoemulsion to enable mammary tissue localization of C6 ceramide.

## 1. Introduction

Breast cancer is one of the most prevalent types of cancer worldwide. It is estimated that approximately 12% of the women will be diagnosed with this disease in their lifetime (Ward et al., 2015; Groen et al., 2017). Among the diseases’ various types, ductal carcinoma *in situ* (DCIS) represents approximately 20% of the mammographically detected breast cancers, an incidence that has increased sharply over the last decades mainly due to the improvement in the screening techniques (Ward et al., 2015). DCIS itself displays a wide range of histological diversity, expressed as grades, and is considered a precursor lesion, with up to 50% of the cases progressing to invasive ductal carcinoma (Sagara et al., 2015). Due to the risk of progression, the current standard of care for all grades of DCIS is surgical excision (breast-conserving or mastectomy) followed by radiation therapy and oral tamoxifen for estrogen-positive tumors (Groen et al., 2017).

However, when it comes to low-grade DCIS, this standard of care has been questioned by several groups, as recent studies have suggested that it does not seem to increase the breast cancer-specific survival for patients with low-grade DCIS at the time of diagnosis, and the rise in DCIS diagnoses (and treatment) has not been accompanied by a corresponding reduction in invasive cancer incidence (Narod et al., 2015; Sagara et al., 2015). As a result, research involving the effect of active surveillance, identification of new and more specific biomarkers and development of novel strategies for localized treatment has been encouraged (Francis et al., 2015a, 2015b; Benson et al., 2016). Considering that development of the lesions start in the mammary ducts, intraductal drug administration emerges as a promising route for local treatment.

The feasibility of this route of administration has been demonstrated for delivery of drugs like paclitaxel, doxorubicin and curcumin in pre-clinical models with chemically induced carcinomas and in transgenic HER-2/neu models, resulting in regression of established tumors and prevention of tumor development (Murata et al., 2006; Chun et al., 2012; Krause et al., 2013). Moreover, cannulation of specific mammary ducts for drug administration as neoadjuvant therapy for breast cancer has been demonstrated to be safe and well tolerated (Love et al., 2009; Mahoney et al., 2013; Zhang et al., 2014). However, these studies employed drug solutions or formulations designed for other administration routes, which do not take into consideration the characteristics and goals of the intraductal route.

This study aims at developing and evaluating a multifunctional bioadhesive nanocarrier for intraductal delivery to increase the concentration of chemotherapeutic drugs at the therapeutic target while reducing systemic exposure to attenuate adverse effects. Our ultimate goal is to develop a new alternative that can be employed for local treatment of low grade DCIS as well as other pre-cancer lesions and benign diseases that increase the risk for development of breast cancer. Here, C6 ceramide was selected as the chemotherapeutic compound; it is a sphingolipid signaling molecule capable of mediating several biological responses and inducing both apoptotic and non-apoptotic cell death (Struckhoff et al., 2004). Rises in ceramide levels in tumor cells have been observed after treatment with vincristine and paclitaxel, and drugs that improve ceramide accumulation seems to increase sensitivity to chemotherapy (Olshefski & Ladisch, 2001; Senchenkov et al., 2001). Treatment of breast cancer cells with short-chain ceramides and 4,6-diene derivatives resulted in induction of apoptosis via the mitochondrial pathway, as demonstrated by release of cytochrome c, loss of membrane asymmetry and a decrease in the membrane potential (Struckhoff et al., 2004; Stover et al., 2005). Moreover, its selective cytotoxicity towards cancer over non-transformed (normal) cells have been reported, which makes C6 ceramide an attractive candidate for treatment of pre-tumor and low grade DCIS lesions that affects small regions due to the possibility of preserving healthy areas of the tissue (Selzner et al., 2001; Lopez-Marure et al., 2002).

To reach the study goal, our strategy was three-fold: (i) to develop multifunction oil-in-water nanoemulsions and assess their bioadhesive potential, safety and kinetics of C6 ceramide release, (ii) to assess the effect of ceramide nanoencapsulation on its toxicity against breast cancer cells, selective effects of the nanoemulsion towards cancer cells, as well as the occurrence of synergism with formulation components, and (iii) to assess whether the bioadhesive properties of the nanoemulsion improve mammary tissue targeting and prolong retention of the drug in the tissue compared to intraductal delivery of a drug solution and systemic drug administration using whole animal *in vivo* imaging.

## 2. Material and methods

### 2.1. Material

Polysorbate 80 (Tween 80), tributyrin, DMSO, chitosan (low molecular weight), porcine mucin, poloxamer 407 and propylene glycol were obtained from Sigma (St. Louis, MO). Tricaprylin was kindly supplied by Abitec Corporation (Janesville, WI), and monoolein from Kerry (Belloit, WI). Acetonitrile, ethanol and methanol were purchased from Mallinckrodt Baker (Phillipsburg, NJ) and C6 ceramide (NBD and plain) from Avanti Polar Lipids (Alabaster, AL).

### 2.2. Design and obtainment of the multifunctional nanocarrier

#### 2.2.1. Components selection

Three components were selected for the oil phase: monoolein, due its bioadhesive properties (Ganem-Quintanar et al., 2000), tricaprylin, for decreasing Ostwald Ripening and improving nanoemulsion droplets stability (Carvalho et al., 2017a), and tributyrin, a prodrug of butyric acid capable of inducing apoptosis in several tumor cell lineages, to potentiate the formulation´s cytotoxic effects (Heidor et al., 2012; Kang et al., 2012; Carvalho et al., 2017b). Polysorbate 80 (Tween 80) was selected as a surfactant due to its general safety and ability to originate nanoemulsions (Hoeller et al., 2009; Nornoo et al., 2009). The aqueous phase was composed of a solution of chitosan and poloxamer 407. Chitosan is a polysaccharide, selected to impart the positive charge and bioadhesive properties to the nanoemulsion, improving its interactions with negatively charged proteins (especially mucin) present in the mammary ducts, and increasing formulation local retention (Mukhopadhyay et al., 2011; da Silva et al., 2016); poloxamer is an amphiphilic polymer capable of increasing nanoemulsion stability due to steric hindrance between oil droplets (Torcello-Gómez et al., 2014).

#### 2.2.2. Obtainment

Monoolein, tributyrin and tricaprylin were mixed at 1:2:1 (monoolein:tributyrin:tricaprylin, w/w/w for a tributyrin content in the nanocarrier of 8.5% w/w) to form the oil phase. For tributyrin-free nanocarriers, used as controls in cytotoxicity experiments to assess synergism with ceramide, the oil phase was composed of monoolein:tricaprylin at 1:3 (w/w). To avoid confusion, when nanocarriers without tributyrin are used, they will be referred to as tributyrin-free nanocarriers (or NE in figures), while the term ‘selected nanocarrier’ will be used to refer to the nanoemulsion containing tributyrin (NE-T in figures).

Oil phase and surfactant were vortex mixed at 1:1 (w/w, Genie 2, Scientific Industries, Bohemia, NY) at room temperature for approximately 30 s, followed by addition of the aqueous phase and probe sonication for 20 min in pulses (58 s on and 30 s off) in an ice bath using 40% maximum amplitude (VCX500, Sonics, Newtown, CT). A critical step to obtain cationic nanoemulsion with adequate size was chitosan incorporation, which was performed according to two protocols: in the first, chitosan solution (1%, w/w) was mixed with a poloxamer solution (2%, w/w) at 1:1 (v/v) to compose the aqueous phase; this was subsequently added to the oil phase-surfactant mixture (66% w/w) and probe-sonicated. In the second protocol, the nanoemulsion was obtained by adding a 1.3% (w/w) poloxamer solution (51% of the final formulation, w/w) to the oil phase-surfactant mixture (1:1, total of 34% of the formulation) followed by probe sonication. Subsequently, the pre-formed nanoemulsion was incubated with the remaining aqueous phase (15% w/w) of the nanoemulsion, composed of a chitosan solution (2% m/m) under magnetic stirring (Corning plate, 100 rpm) for 30 minutes at room temperature. As control, nanocarriers without chitosan (aqueous phase composed of poloxamer solution at 1%) were also prepared. Based on the properties of the resulting nanoemulsion (see ‘Results’ section), the first protocol was selected.

### 2.3. Characterization of the nanoemulsions

Size and zeta potential were determined using Zetasizer NanoZS equipment (Malvern, UK) after dilution with water at 1:100 (w/w). Size and zeta potential of three samples from different batches were measured. These parameters were measured immediately after obtainment of the formulation (initial values) and after drug encapsulation to assess the influence of C6 ceramide on the physicochemical properties of the nanoemulsion. Rheological behavior was assessed with a R/S Plus controlled stress rheometer with RC75-1 cone (Brookfield Engineering laboratories, Middleboro, MA), and a bath circulator for temperature control, set at 37 °C. The experiments were performed in duplicate, with shear rates up to 3000 s^−1^. The relationship between the shear stress and the shear rate of each formulation was evaluated using the Power law equation τ = K γ^n^, where *τ* is the shear stress, *γ* is the rate of shear, *K* is the consistency index and *n* is the flow index (Hosmer et al., 2013).

### 2.4. Evaluation of the bioadhesive potential of the formulation

As an index of the bioadhesive potential of the formulation, the interaction between the nanoemulsion and mucin was assessed (Mazzarino et al., 2012). A dispersion of porcine stomach mucin at 250 µg/mL was prepared in phosphate buffer (Sigma, St. Louis, MO, 10 mM, pH 7.4) and incubated with the nanoemulsion (1%, v/v) at 37 °C for 30 min under stirring (100 rpm, *n* = 3 for each treatment) (Mazzarino et al., 2012; Andreani et al., 2015). As controls, the mucin dispersion and the nanoemulsion were incubated individually with PBS.

After incubation, samples were diluted (1:100 v/v) in water for assessment of size and zeta potential. Occurrence of nanoemulsion-mucin interactions was indicated by an increase in nanodroplets size and inversion of zeta potential as expected from droplet coating with mucin (da Silva et al., 2016).

### 2.5. Irritation potential and biosafety

#### 2.5.1. HET-CAM

Hen’s Egg Test – Chorioallantoic Membrane (HET-CAM) was used to estimate the irritation potential of the unloaded nanoemulsion to the administration site following previously published guidelines (INVITOX protocol 96) (McKenzie et al., 2015; Contri et al., 2016). Fertilized chicken eggs were obtained from Sabor Natural (São Paulo, SP, Brazil) and incubated for 9 days at 37 °C and 55% humidity in incubators (Premium Ecologica, Belo Horizonte, MG, Brazil) with automatic rotation every 2 h. Following photodocumentation of the chorioallantoic membrane (Nikon, SMZ 1500, Tokyo, Japan), the nanoemulsion (100 mg) was applied for 5 min; NaCl 0.9% and NaOH (0.1 M) were used as negative and positive controls, respectively. During this period of time, the membrane was assessed for coagulation, hemorrhage and lysis as previously described (Contri et al., 2016). Each treatment was performed in 4–6 eggs.

#### 2.5.2. Galleria mellonella model

A safety assay using *Galleria mellonella* larvae was employed to assess the toxicity of the nanoemulsion prior to its administration in rats. The larvae (2.0–2.5 cm of length and 150–200 mg of body weight) were separated in seven groups (6 treatments + control) with 16–20 larvae each group. Larvae was allocated in Petri dishes containing beeswax and pollen and maintained at 37 °C. Each group received the nanoemulsion diluted at 0.5, 2, 10, 50, 250 or 500 mg/mL in PBS, while PBS alone was used as vehicle control; 10 µL of sample was injected in the last larvae proleg. Survival was assessed every 24 h during five days for survival curve plotting; greater survival is an indication of low toxicity and, therefore, increased safety of formulation.

### 2.6. C6 ceramide encapsulation and *in vitro* release

C6 ceramide was added into the oil phase-surfactant mixture of the selected nanocarriers to obtain a final concentration of 0.4% (w/w). For *in vitro* release and *in vivo* studies (section 2.8), NBD ceramide was used, whereas the plain compound was employed to assess the cytotoxicity of the nanocarrier against cells in culture (section 2.7). The mixtures were bath sonicated (Quimis, Diadema, SP, Brazil) for 20 min to dissolve ceramide before addition of the aqueous phase.

Considering that the nature of the formulations might hinder the release and bioavailability of lipophilic drugs (Lopes et al., 2009), we assessed C6 ceramide release from the nanoemulsion using Franz diffusion cells (diffusion area of 1.77 cm^2^; Hanson, Chatsworth, CA) and a cellulose membrane. The receptor phase, consisting of phosphate buffer with 20% ethanol, was maintained at 37 ± 0.5° C with magnetic stirring at 350 rpm throughout the experiment (Pepe et al., 2012; Cichewicz et al., 2013; Carvalho et al., 2017 b). Treatment was performed by transferring the volume equivalent to 100 mg of the nanocarrier after determining the relationship between mass and volume of the formulation (*n* = 4–5). Samples of the receptor phase were collected at 2, 4, 6, and 8 h post-application, filtered through 0.45 μm pore membranes, and assayed for the drug. For initial time points, the receptor phase was concentrated four times using a vacuum concentrator (Concentrator plus 5301, Eppendorf, Hamburg, Germany) before drug assay. C6 ceramide delivery was quantified using fluorimetry in a plate reader (Biotek Synergy HT, Winooski, VT, USA), and 485/530 nm as excitation/emission wavelengths. Calibration curves in the range of 0.2–20.0 μg/mL were used, and coefficients of determination ≥0.990 were obtained (Carvalho et al., 2017 b). The release rates were calculated from the slope of the linear portion of the plots of cumulative drug released against time (Rozman et al., 2009; Ng et al., 2017).

### 2.7. *In vitro* cytotoxicity assay

Cytotoxicity assays were conducted with three goals in mind: (i) to assess the effect of nanoencapsulation on ceramide cytotoxicity against cancer cells; (ii) to evaluate whether inclusion of tributyrin in the nanocarrier potentiated C6 ceramide cytotoxicity, and (iii) to assess the selectivity of cytotoxic effects against cancer over ‘normal’, non-transformed cells.

Breast cancer cells (MCF7) and human retinal epithelium cells (RPE, ‘normal’, non-transformed cell) were obtained from ATCC, and cultured in DMEM - GlutaMAX™ (Gibco, Carlsbad, CA) medium supplemented with 10% fetal bovine serum (FBS, Gibco, Carlsbad, CA), penicillin (100 U/mL, Gibco, Carlsbad, CA) and streptomycin (100 μg/mL Gibco, Carlsbad, CA). Cells were grown at 37 °C and 5% CO_2_ atmosphere, and cell passage was performed at 80% confluence.

For assessment of viability, cells were incubated with the MTT solution (0.5 mg/mL) for 3 h, and the formazan product was extracted with DMSO and absorbance was quantified with spectrophotometer (Multiskan™ FC Microplate Photometer, Thermo Scientific, Waltham, MA) set at 595 nm. DMSO-treated cells and cells treated with doxorubicin (0.02–9.2 μM) were used as negative and positive control, respectively. Experiments were performed in triplicate and repeated 3–5 times.

#### 2.7.1. Influence of tributyrin concentration on nanoemulsion cytotoxicity

In this experiment, the influence of tributyrin on the nanocarrier cytotoxicity was assessed by treating cells with the unloaded (without ceramide) nanocarrier containing 0 or 8.5% of tributyrin. The tributyrin-free nanocarriers and those containing tributyrin at 8.5% were diluted in culture medium within the range 0.01–64 mg/mL.

#### 2.7.2. Influence of nanoencapsulation and presence of tributyrin on the cytotoxicity of C6 ceramide against cancer cells

Our goals in this experiment were to assess the effect of nanoencapsulation on C6 ceramide cytotoxicity, and whether the presence of tributyrin in the formulation potentiates drug cytotoxicity. Cells were treated for 24 h with C6 ceramide solution in DMSO (0.06–240 μM), or ceramide-loaded nanoemulsions that were either free of tributyrin or containing this triglyceride at 8.5% (0.16–20 μM). After treatment, cell culture medium was replaced by treatment-free medium, and cells were incubated for additional 21 h before MTT assay to ensure that the drug had enough time to act as described for other cytotoxic agents (Desai et al., 2008; Hosmer et al., 2013). The concentration of C6 ceramide and tributyrin necessary to reduce cell viability to 50% (EC_50_) was estimated by fitting the average viability curve for each treatment as a function of drug concentration with polynomial models (using Orign 9.0, OrignLab Corporation, Wellesley Hills, MA) (Carvalho et al., 2017b). The EC_50_ values were used to obtain the combination index (CI), calculated to assess synergism of tributyrin and C6 ceramide when used in combination according to the equation of Chou-Talalay (Chou, 2010) as previously described (Carvalho et al., 2017 b):

(1) CI=Ca,50/ECA+Cb,50/ECB

where Ca,50 and Cb,50 are tributyrin and ceramide concentrations necessary to reduce cell viability to 50% (EC_50_) were used in combination, ECA represents tributyrin EC_50_ when administered as a single agent, and ECB represents ceramide EC_50_ when administered as a single agent. This equation offers quantitative definition for additive effect (CI =1), synergism (CI <1), and antagonism (CI >1) in drug combinations.

#### 2.7.3. Nanoemulsion cytotoxicity against non-transformed cells

To assess whether nanoencapsulation affects C6 ceramide selective effects towards cancer cells, RPE cells were treated with the unloaded nanoemulsion, C6 ceramide-loaded formulation and drug solution as described in section 2.7.2. Because of the synergistic effect observed for tributyrin and C6 ceramide (see ‘Results’ section), this assay was conducted with the nanoemulsion containing tributyrin at 8.5%.

### 2.8. *In vivo* intraductal administration, histological changes and mammary tissue targeting

This experiment was conducted with two main goals: (i) to compare C6 ceramide localization into the mammary tissue after intraductal and systemic (i.p.) administration of the nanocarrier, and (ii) to evaluate the ability of the bioadhesive nanoemulsion to improve the residence time of ceramide in the mammary tissue compared to a drug solution. Female Wistar rats were housed in the Department of Pharmacology animal facility with free access to food and water until they reached 250 ± 20 g. The animal room was kept under a 12:12 h light–dark cycle (lights on at 7:00 am), and temperature was maintained between 22 and 23 °C. The protocol was conducted in accordance with the guidelines from the Brazilian Council for Control of Animal Experimentation (CONCEA), and approved by the Animal Care and Use Committee at the Institute of Biomedical Sciences of the University of São Paulo (protocol number 69/2016, São Paulo, Brazil).

Briefly, rats were anesthetized with isoflurane (Cristalia, Itapira, Brazil) and the hair from the abdomen was removed using Veet^®^ cream. Twenty four hours after hair removal, the animals were inspected for signs of skin irritation, and divided in 5 groups (*n* = 3–4/group) based on the treatment: intraductal saline, intraductal unloaded nanoemulsion, intraductal C6 ceramide-loaded nanoemulsion, intraductal C6 ceramide solution in PBS:propylene glycol (2:3 v/v) and i.p. C6 ceramide-loaded nanoemulsion.

For intraductal injection, animals were anesthetized with isoflurane, and the nipples were gently rubbed with gauze soaked in alcohol to remove keratin plugs and reveal the duct orifice (Chun et al., 2012; Krause et al., 2013). Rats have six pairs of nipples and mammary glands along two mammary chains, and unlike humans, each mammary gland possesses a single ductal system. Thus, with a single intraductal injection into each nipple, the formulation has access to the whole ductal tree. We selected three pairs of nipples according to their ease of access for administration of the formulation and control solutions. Under a dissection microscope, 20 μL of the drug solution or the nanoemulsion was injected into the orifice using a 33 G needle attached to a Hamilton syringe (Hamilton, Bonaduz, Switzerland); saline injection was used as control to assess any local damage induced by the nanoemulsion. The accuracy of the administration technique was assessed by injecting a solution of Evans blue vital dye (2% w/v) into the nipples, and ductal tree staining, signs of duct overfilling, ductal wall perforation and presence of edema (indicating injection into the mammary fat pad) were checked (Krause et al., 2013).

Another group of animals received an i.p. injection of the ceramide-loaded nanoemulsion for assessment of mammary tissue targeting after systemic drug administration. Following administration, whole body images were obtained after 2, 24, 48 and 120 h using a bioimaging system (IVIS Spectrum System, Perkin-Elmer Life Sciences, Waltham, MA) to verify drug targeting to the mammary tissue and systemic distribution. The following instrument settings were used for comparison among different groups: exposure time =  5 s, binning factor =  8, excitation/emission =  465/540 nm.

In order to evaluate architectural tissue changes or local damage resulting from the administration of the formulation, histological analysis was performed. Mammary glands were excised 120 h post-injection, fixed in 10% buffered neutral formaldehyde and processed for inclusion in paraffin (Murata et al., 2006). Histological sections of 5 μm were stained with hematoxylin/eosin and microscopically analyzed for their morphology.

### 2.9. Statistical analyses

The results are reported as means ± SD. Data were statistically analyzed using the ANOVA test followed by Tukey post-hoc test (GraphPad Prism software). *Galleria mellonella* larvae survival data was compared pairwise (NE at each concentration *versus* PBS) using log-Rank (Statistica software, version 8). Values were considered significantly different when *p* < .05.

## 3. Results

### 3.1. Obtainment of the multifunctional nanocarrier

**Figure 1.**
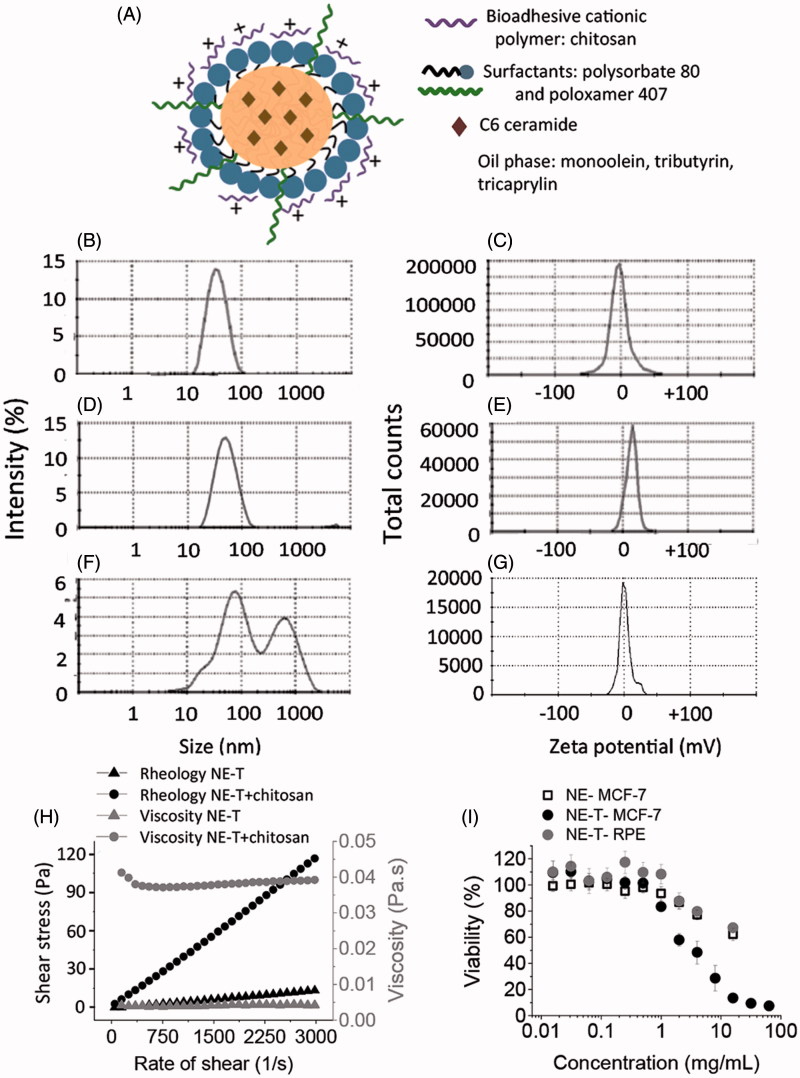
Development and characterization of nanoemulsions. Schematic representation of the loaded nanoemulsion (A), size (B) and zeta potential (C) of formulations obtained without chitosan, size (D) and zeta potential (E) of nanoemulsions obtained with chitosan according to the first protocol (in which chitosan was mixed in the aqueous phase), size (F) and zeta potential (G) of pre-formed nanoemulsions after incubation with chitosan solution (second protocol); all formulations contain tributyrin, and representative graphs resulting from analysis of three formulations from different batches are depicted in these panels. (H) Rheological behavior and viscosity of nanoemulsions with and without chitosan; data shown as average of two samples from different batches. (I) Influence of tributyrin concentration when added into the oil phase on the nanocarrier cytotoxicity against MCF-7 and RPE-cells cells; data shown as average ± standard error, *n* = 9–12.

The first goal of this study was to develop a cationic oil-in-water nanoemulsion with bioadhesive potential, a key feature to optimize localization of the nanocarrier in the mammary duct, and therefore, enhance efficacy in target cells while limiting systemic exposure (Murata et al., 2006). We established ideal physical–chemical parameters based on the administration route: droplet size inferior to 100 nm to avoid any ductal obstruction, PDI <0.25 to ensure homogeneous size distribution, and positive zeta potential to increase retention by electrical interaction with negatively charged proteins (such as mucin) expressed in the duct (Mukhopadhyay et al., 2011; Mazzarino et al., 2012). Figure 1(A) shows a schematic representation of the nanocarrier.

As depicted in Figure 1(B,C), nanocarriers obtained with tributyrin at 8.5% without chitosan displayed nanometric size (39.3 ± 0.8 nm) and negative zeta potential (−2.9 ± 3.5 mV). Slightly larger nanocarriers (46.3 ± 0.7 nm) with positive charge (+13.6 ± 0.3 mV) were obtained when chitosan was incorporated into the aqueous phase prior to nanoemulsion sonication (first protocol, Figure 1(D,E)), whereas incubation of pre-formed nanocarriers with the chitosan solution (second protocol, Figure 1(F,G)) resulted in larger, polydisperse particles (PDI= 0.53 ± 0.10) with a zeta potential close to neutrality (−0.6 ± 0.8 mV). These results demonstrate that the protocol for chitosan addition is critical. Based on these results, the first protocol (in which chitosan was mixed in the aqueous phase before sonication) was selected. Nanocarriers obtained without tributyrin in the oil phase (which was composed of monoolein:tricaprylin at 1:3, w/w) also displayed nanometric size (54.0 ± 3.2 nm) and a positive zeta potential (+7.4 ± 1.8 mV); these nanocarriers were employed in cytotoxicity experiments as controls.

The nanoemulsions displayed Newtonian Behavior independent on chitosan presence as demonstrated by linear relationships between rate of shear and shear stress (Figure 1(H)), and values of the flow index ∼1. Flow is considered Newtonian when *n* = 1, whereas *n* > 1 or *n* < 1 indicates shear-thickening or shear-thinning, respectively (Hosmer et al., 2013). Even though the rheological behavior was similar, the final viscosity of the nanoemulsion, determined by averaging viscosity values at individual rates of shear, increased approximately 9.5-fold with chitosan addition.

### 3.2. Effect of tributyrin concentration on the nanocarrier cytotoxicity

Ideally, nanocarriers should improve the cytotoxicity of anti-tumor drugs mainly against cancer cells. Because tributyrin has been described to affect the viability of melanoma cell lines (Kang et al., 2012; Carvalho et al., 2017 b), we evaluated the effect of its addition on nanocarrier cytotoxicity against breast cancer (MCF-7) and normal, non-transformed (RPE) cells. We acknowledge the functional differences between RPE and cells from the mammary tissue, but since these are epithelial cells, they were selected to mimic possible effects at the healthy epithelial lining of the ducts.

Viability of MCF-7 cells was reduced as the nanocarrier concentration in the medium increased, and the tributyrin-containing nanoemulsion became more cytotoxic than that without tributyrin in a concentration-dependent manner (Figure 1(I)). Viability was higher than 50% when MCF-7 cells were treated with the tributyrin-free nanoemulsion at concentrations up to 16 mg/mL, while inclusion of tributyrin decreased the nanocarrier EC_50_ to 2.9 mg/mL. On the other hand, treatment of RPE cells with the tributyrin-containing nanocarrier at concentrations up to 16 mg/mL resulted in viabilities higher than 50%, which demonstrate that tributyrin addition affects cancer cells in a more pronounced manner, and prompted us to select the tributyrin-containing nanoemulsion for further studies.

### 3.3. Evaluation of the bioadhesive potential of the nanocarriers

**Figure 2.**
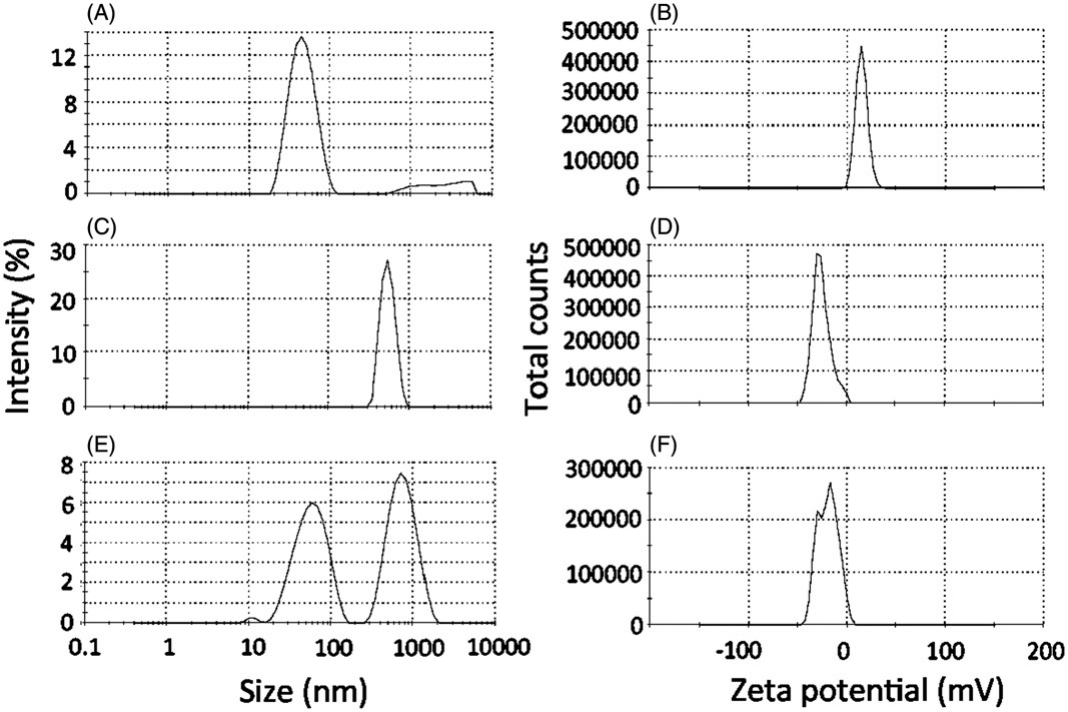
Bioadhesive properties of the optimized nanoemulsion as demonstrated by changes on size and zeta potential. (A, C, E) Size of aggregates resulting from the incubation of the nanoemulsion with PBS (A), mucin dispersion with PBS (C) or nanoemulsion with the mucin dispersion (E). (B, D, F) Zeta potential of aggregates resulting from the incubation of the nanoemulsion with PBS (B), mucin dispersion with PBS (D) or nanoemulsion with the mucin dispersion (F). The figure shows representative graphs, *n* = 3.

To assess the bioadhesive potential of the nanocarrier, it was incubated with a dispersion of mucin, and changes on size and zeta potential were evaluated. As controls, mucin dispersion and the nanoemulsion were incubated separately with PBS. Incubation of the nanoemulsion with PBS did not result in pronounced changes on its size or zeta potential (47.3 ± 0.8 nm, +13.2 ± 2.7 mV, Figure 2(A,B)). After incubation with PBS, the mucin dispersion displayed negatively charged aggregates (−31.1 ± 1.6 mV) of around 650 nm (Figure 2(C,D)). Incubation of the nanoemulsion with mucin resulted in two populations of aggregates with diameters of approximately 60 and 708 nm, which most likely reflect the co-existence of the nanoemulsion droplets with mucin aggregates (Figure 2(E)). Considering the increase on nanoemulsion droplet size from 47.3 (after incubation with PBS) to 60 nm (after incubation of mucin), it is reasonable to suggest the occurrence of interactions between the polysaccharide and nanoemulsion droplets. This is further evidenced by the zeta potential shift from positive (+13.2 ± 2.7 mV) to negative values (peaks at −30.0 and −12.2 mV, Figure 2(F)) after incubation with mucin, demonstrating the change on charge distribution at the nanodroplets surface. These interactions support the bioadhesive potential of the nanoemulsion.

### 3.4. Irritation potential and safety

**Figure 3.**
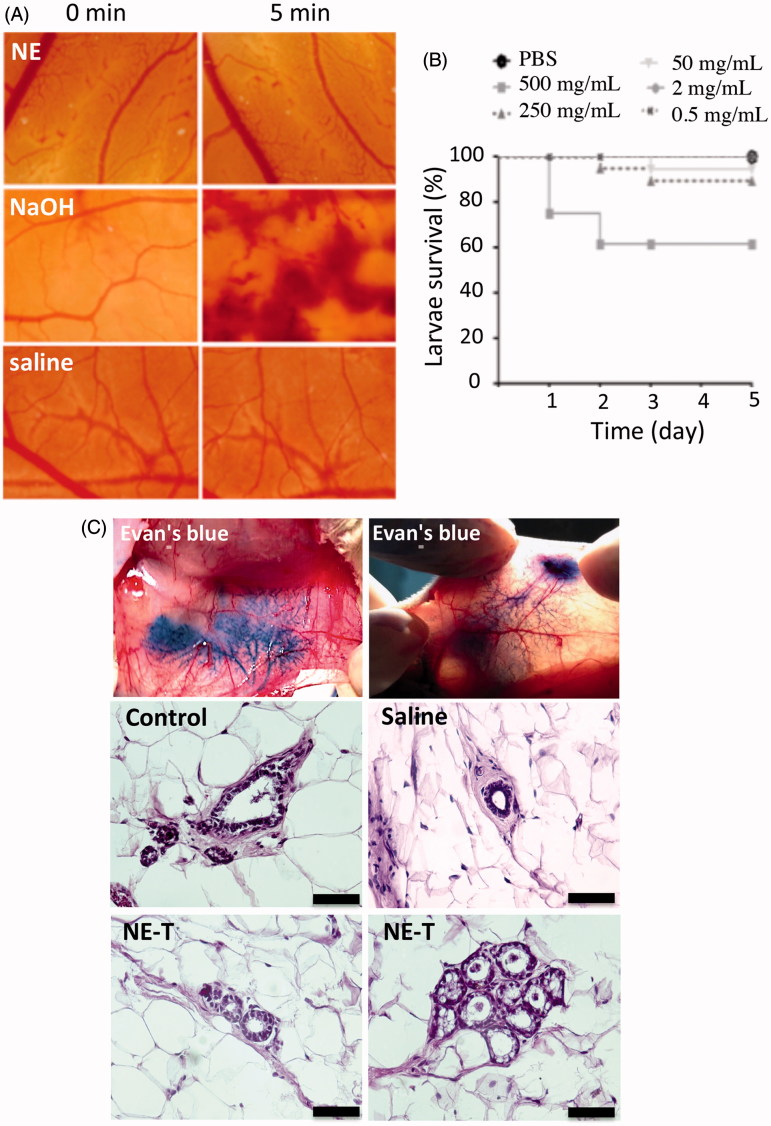
Irritation potential and toxicity of the optimized nanoemulsion. (A) Signs of irritation (coagulation, lysis and hemorrhage) on the chorioallanthoic membrane as a function of time resulting from application of the nanoemulsion in comparison with saline (negative control) and NaOH (positive control); representative images from experiments performed with 4–6 eggs. (B) Survival of *Galleria mellonella* as a function of time after injection of increasing concentrations of the nanoemulsion (0.5–500 mg/mL). (C) Influence of nanocarrier treatment on the histological characteristics of mammary tissue. The top two panels depict ductal tree staining after intraductal administration of an Evan’s blue solution to demonstrate that the injection is in the correct area; the others represent cross-section of the mammary tissue from untreated animals, or those treated with saline or the unloaded selected nanoemulsion (NE-T). Bar= 50 μm, *n* = 3–4/group.

The finding that the selected nanoemulsion (containing tributyrin) displays some cytotoxicity against normal cells (although less pronounced compared to cancer cells) prompted us to assess the nanoemulsion potential for causing irritation to the administration site using HET-CAM. The underlying principle of the method is the measurement of time-dependent occurrence of vascular toxicity endpoints (hemorrhage, lysis and coagulation) on the chorioallontoic membrane when exposed to a test sample. Initially employed to assess eye irritation, this model has now been used to assess irritation to the skin and other tissues following parenteral administration (Mehling et al., 2007; Eichenbaum et al., 2013). Formulation effects on the chorioallontoic membrane for 5 min are represented in Figure 3(A). As expected, application of the saline solution to healthy membranes produced no perceptible change over the five-minute time window. In accordance with previous observations, NAOH (positive control) caused lysis, coagulation and severe hemorrhage during the studied time period, resulting in a score of 17.1 ± 0.4, which classifies this solution as severe irritant (McKenzie et al., 2015; Fangueiro et al., 2016). In contrast, the nanoemulsion treatment resulted in few points of lysis after approximately 5 min, resulting in a calculated score of 0.03 ± 0.02, which classifies the formulation as nonirritant. According to these results, we do not expect that the nanoemulsion intraductal injection cause irritation at the administration site.

Even though we aim for a local drug delivery, we must ensure formulation safety in case part of the nanoemulsion is systemically absorbed. To evaluate nanoemulsion systemic toxicity, a safety assay using *Galleria mellonella* larvae was employed. This is considered an alternative model that displays several advantages as a screening system, including the larvae ability to survive temperatures over 30 °C, and the possibility of sample administration via three different routes (topical, oral or proleg injection), allowing desired *in vivo* routes to be matched in the model (Megaw et al., 2015; Maguire et al., 2016). Here we used proleg injection to mimic systemic absorption. As expected, the toxicity of the formulation was dose-dependent; compared to PBS (vehicle control), significant decreases (*p* < .001) on larvae survival were observed only when the highest concentration of the nanoemulsion was administered (500 mg/mL, Figure 3(B)). Because larvae survival was higher than 50% at all tested concentrations, the dose considered lethal for 50% of the insects (LD_50_) is estimated to be higher than 500 mg/mL.

Representative histological pictures of the mammary tissue of untreated rats (control) or animals treated with saline or the unloaded nanoemulsion are depicted in the panel C of Figure 3. First, it is possible to note that intraductal administration filled the whole mammary tree, as can be observed in the top panels of Figure 3(C) after injection of a dye solution, which indicates that the administration technique is effective. Control, non-injected ducts are characterized by a layer of cuboidal epithelial cells, embedded in stroma and surrounded by adipose tissue, referred to as the fat pad (Masso-Welch et al., 2000). A very similar architecture was observed in tissue sections from animals treated with saline or the unloaded nanoemulsion (NE-T). Alveoli from tissue treated with the unloaded nanoemulsion (NE) are also represented in Figure 3(C) to confirm the presence of typical structures. Tissue integrity and absence of histological alterations and inflammatory cell infiltrates suggest that the formulation did not cause tissue damage, and thus, its intraductal administration can be considered safe.

### 3.5. Ceramide incorporation and in vitro release

Incorporation of ceramide at 0.4% (w/w) did not promote significant (*p* > .05) changes in the size (52.1 ± 4.1 nm) or zeta potential (+11.5 ± 0.9 mV) of the nanocarrier. Considering the high drug lipophilicity (logP >4) and affinity of these types of compounds for the oil phase of emulsified nanocarriers (Nybond et al., 2005; Pepe et al., 2013; Thomas et al., 2014), one of our concerns was drug retention within the formulation for long periods of time, which could compromise efficacy. Thus, *in vitro* studies were performed to assess the kinetics of drug release (Supplementary Figure 1). At the longest time point studied (24 h), approximately 32% (∼128 μg/cm^2^) of ceramide was released from the nanoemulsion, and a linear relationship was obtained when cumulative ceramide release was plotted as a function of time (*r* = 0.993) during the time window investigated, suggesting that release follows zero-order kinetics. Similar kinetics has been observed for other nanoemulsion-based systems (Tayel et al., 2013; Zhang et al., 2013). It is worth mentioning that the longest time point investigated was 24 h due to the low solubility of the drug in aqueous-based receptor phases, as previously reported (46.8 ± 9.4 μg/mL) (Carvalho et al., 2017 b). Drug concentration in the aqueous phase did not exceed 40% of its solubility to avoid constraining dissolution and release, which could lead to underestimation of release (Adachi et al., 2015).

### 3.6. Effect of nanoencapsulation on C6 ceramide cytotoxicity

**Figure 4.**
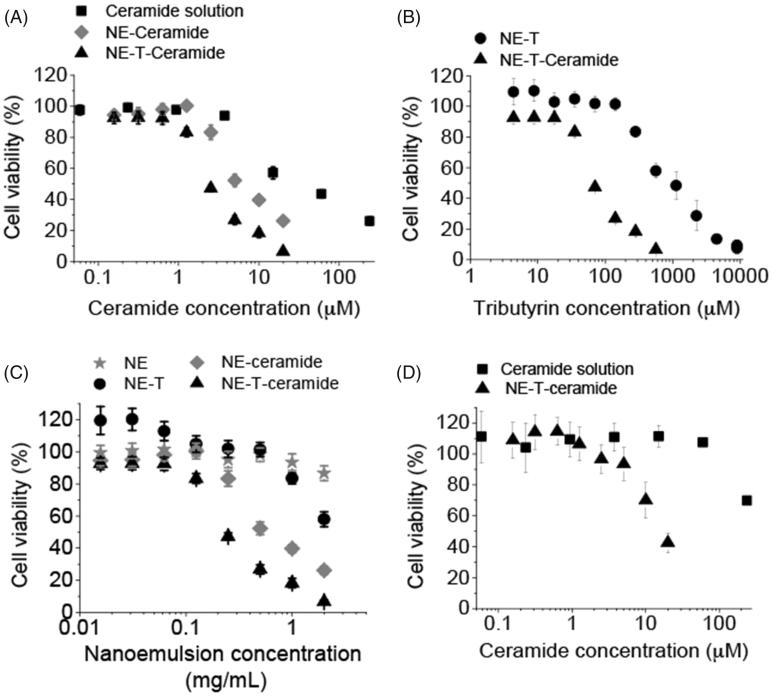
Cytotoxicity of the nanoemulsion (NE) and C6 ceramide. (A) influence of ceramide nanoencapsulation in nanocarriers without (NE) or with tributyrin (NE-T) on the cytotoxicity against MCF-7 cells, (B) influence of tributyrin incorporation and association with ceramide on nanocarrier cytotoxicity against MCF-7 cells; (C) comparison of nanocarrier cytotoxicity with and without ceramide and tributyrin, (D) influence of ceramide nanoencapsulation in nanocarriers without (NE) or with tributyrin (NE-T) on the cytotoxicity against RPE cells. Experiments were performed in triplicate and repeated 3–5 times; data shown as average ± standard error.

The cytotoxicity of the C6 ceramide solution was dose dependent, producing significant (*p* < .05) decreases in cell viability when used at 15 μM and higher (Figure 4(A)). Compared to the drug solution, encapsulation into the tributyrin-free nanoemulsion promoted a viability curve shift to the left, with lower viabilities observed when cells were treated with the nanoencapsulated C6 ceramide. More specifically, the concentration of C6 ceramide necessary to reduce cell viability to 50% (EC_50_) was 4.5-fold lower when it was encapsulated in the tributyrin-free nanocarrier in comparison with its DMSO solution (Figure 4(A) and Table 1). Presence of tributyrin at 8.5% in the nanoemulsion further decreased the EC_50_ of ceramide (2.6-fold). These results demonstrate that incorporation in the nanoemulsion increases C6 Ceramide cytotoxicity, and that presence of tributyrin potentiates this effect.

**Table 1.** Concentrations of C6 ceramide and tributyrin necessary to reduce cell viability to 50% (EC_50_).

Formulation	EC_50_ (μM)
MCF7 cells	
C6 Ceramide solution	29.0
C6 Ceramide in NE	6.4
C6 ceramide in NE-T	2.5
Tributyrin in NE-T	890.0
Tributyrin in NE-T + C6 ceramide	79.7
RPE cells	
C6 ceramide solution	>240
C6 ceramide in NE-T	14.7

Determining cell viability as a function of tributyrin concentration allowed us to calculate the EC_50_ for the triglyceride in the presence and absence of ceramide. EC_50_ values were also reduced in the presence of ceramide (approximately 11-fold, Figure 4(B)), suggesting that combination of tributyrin and C6 ceramide in the nanocarrier is advantageous for potentiation of cytotoxicity. This effect can be better visualized in Figure 4(C), which shows a comparison of ceramide C6-unloaded and loaded nanoemulsion. Taking a concentration of 0.25 mg/mL of the nanocarrier for example, cell viability was 95% using tributyrin-free nanoemulsion, 101 and 83% when either tributyrin or ceramide were added, and dropped to 47% when both compounds were co-encapsulated.

The fact that the combination of two compounds resulted in more pronounced cytotoxic effects compared to their effects separately does not necessarily indicate synergism; additive effects are possible (Chou, 2010). To demonstrate synergism, the nature of interaction between C6 ceramide and tributyrin was further evaluated by calculating the combination index (CI) using Equation 1, previously introduced by Chou (2010) to quantitatively depict synergistic (CI <1), additive (CI =1), and antagonist effects (CI >1). Since the calculated combination index was 0.48, a synergistic effect can be assumed.

It is described in the literature that apoptosis mediated by C6 ceramide is more pronounced against cancer cells while healthy tissues are shown to be less affected (Stover et al., 2005). Therefore, we evaluated whether the selective cytotoxicity is also observed when C6 ceramide is incorporated in the selected (containing tributyrin) nanoemulsion. Similarly to what we observed for transformed cells, nanoencapsulation of C6 ceramide in the selected nanocarrier promoted a shift to the left of the viability curve, demonstrating increased cytotoxicity. However, the estimated EC_50_ of ceramide when incorporated in the selected nanoemulsion was 14.7 μM (Table 1), which is over 5-fold higher than that observed for MCF-7, and demonstrates a more pronounced cytotoxicity against these cancer cells.

### 3.7. *In vivo* intraductal administration and mammary tissue targeting

**Figure 5.**
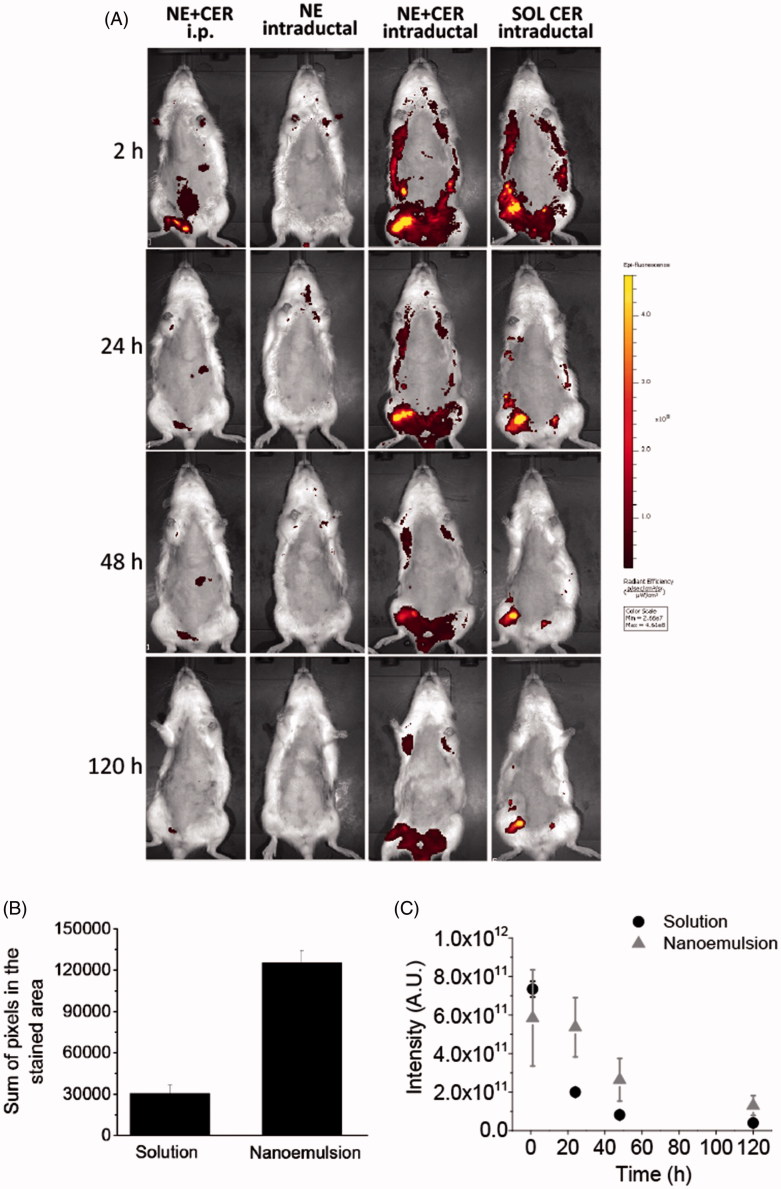
Tissue localization and distribution of C6 ceramide. (A) Representative *in vivo* whole body images of animals treated with the nanocarrier or a control solution as a function of time; ceramide-loaded nanoemulsion containing tributyrin (NE-T + CER) was administered using the intraperitoneal or intraductal routes. Intraductal administration of the unloaded nanoemulsion was used as control for nanocarrier autofluorescence, whereas ceramide solution was administered intraductally to compare retention time. The figure shows representative images from experiments conducted with 3–4 animals. (B) sum of pixels from the regions stained with C6 ceramide at 120 h post intraductal administration of a ceramide solution or the optimized nanoemulsion. (C) fluorescence decay as a function of time after intraductal administration of a ceramide solution or the optimized nanoemulsion.

Figure 5 depicts representative images of animals subjected to systemic or intraductal administration of the nanocarrier or controls. As evident from Figure 5(A), i.p. administration of the nanoemulsion loaded with the fluorescently labeled C6 ceramide resulted in fluorescent staining of the abdominal cavity, and some fluorescence at the mammary tissue region 2 h after administration. Extremely low fluorescence staining was observed at 24 h and afterwards, suggesting fast drug distribution and elimination. Intraductal administration of the unloaded nanoemulsion resulted in no fluorescent staining at the mammary tissue, suggesting that formulation-associated fluorescence or tissue autofluorescence are low and do not interfere with the experiment.

Compared to the intraperitoneal injection, a much more pronounced mammary retention of ceramide was observed after intraductal administration of the nanoemulsion, which is indicated by the stronger fluorescent mammary tissue staining. Drug localization in the mammary tissue was evident at 2 h post-application of the nanoemulsion, and even though fluorescence signals decreased with time, staining of large areas (that correspond to the mammary tissue) was still observed 120 h after administration. Administration of the ceramide solution also resulted in drug localization in the mammary tissue at 2 h post-application, but fluorescence signals decreased faster and in a more pronounced manner within 120 h compared to the nanoemulsion, and the regions showing fluorescence staining were smaller at the last time point. To better compare the size of regions showing fluorescence in animals treated with the nanoemulsion and the control solution, a pixel count in the fluorescent areas was performed using Photoshop after conversion of the fluorescent regions to black areas. At 120 h, the sum of pixels in the stained areas was an average of 4.2-fold larger in nanoemulsion-treated rats compared to the solution (Figure 5(B)). Additionally, prolonged fluorescence staining of the mammary area was observed in the nanoemulsion-treated animals within the time period studied, as demonstrated by the slower fluorescence intensity decay (Figure 5(C)). These results demonstrated that intraductal administration provides mammary tissue targeting, with the nanoemulsion providing longer, improved ceramide retention.

## 4. Discussion

Intraductal administration of drugs for treatment of low grade DCIS and other pre-cancer lesions and diseases that increase the risk for breast cancer represents a new strategy to combine efficacy and reduction of systemic adverse effects frequently observed in cancer treatment. Local approaches have been successfully used in other types of cancer, as for example in bladder cancer, in which intravesical treatment is used as adjuvant therapy after surgical transurethral resection (Shen et al., 2008). However, in spite of the growing interest in intraductal administration, development of formulations for this route has been very slow, with only one study focusing on PEG-based nanocarriers, and the influence of aggregate size on local retention (Singh et al., 2012). The present study contributes to fill this gap.

Previous studies have suggested that small drugs are likely to diffuse fast into the systemic circulation after intraductal administration, and thus, localization in the mammary tissue can be optimized by ductal retention strategies (Singh et al., 2012). Based on these observations, we developed surface-modified multifunctional nanocarriers using the bioadhesive cationic chitosan to improve local retention. The multifunctional aspect was provided by the multiple functions of the selected components: tributyrin played the dual role of increasing the cytotoxicity of the nanocarrier, especially against tumor cells, and composing the oil phase, while chitosan promoted bioadhesive properties and increased the viscosity of the formulation. This viscosity increase is interesting because it contributes to improve formulation residence time and thus, benefit drug transport (Valenta & Schultz, 2004; Biruss & Valenta, 2008; Phelps et al., 2011). In spite of the viscosity increase, the Newtonian rheological behavior was not altered; as viscosity of Newtonian systems is not expected to change due to shearing, the force necessary for injection will depend only on the injecting speed and formulation viscosity, parameters that can be controlled during administration (Allahham et al., 2004). This flow behavior has been previously reported for other micro- and nanoemulsions, and confirm the general concept that diluted dispersions and suspensions tend to approach Newtonian behavior because interactions among particles are reduced by dilution (Allahham et al., 2004; Krahn et al., 2012; Carvalho et al., 2017a).

Ideally, nanocarrier-based antitumor strategies should display stronger toxicity against cancer cells while preserving the integrity of healthy tissues (Naz et al., 2017; Xiao et al., 2017). Even when targeting is possible, healthy areas in the administration site should be safeguarded, especially in the case of small lesions that do not largely compromise the tissue architecture such as atypical and pre-tumor breast lesions and low grade DCIS (Benson et al., 2016; Akram et al., 2017). To ensure safety to healthy areas, we assessed the cytotoxicity of the unloaded nanoemulsion against non-tumor cells, and its irritation potential in HET-CAM models and *in vivo*. Less pronounced reductions on the viability of normal cells compared to tumor cells were observed, even when tributyrin was included. We have previously observed that its incorporation in nanocarriers affect cytotoxicity on a manner that depended on the type of cancer cell and mutations present (Carvalho et al., 2017 b), but its ability to selectively enhance cytotoxicity against tumor cells is novel and further suggests the use of this formulation for prevention strategies in addition to treatment. Consistent with this finding, the selected nanocarrier (without ceramide) caused no irritation at the site of administration, as evidenced by the low irritation score in HET-CAM models and lack of histological changes in the mammary tissue. These results also corroborate previous observations concerning the applicability of HET-CAM as an alternative method to assess irritation to the administration site (Eichenbaum et al., 2013).

Even though tissue localization is intended, it was necessary to ensure safety in case absorption of a fraction of the formulation occurred. The nanocarrier lethal dose for 50% of *G. mellonella* larvae (LD_50_) could not be determined at the concentration range studied due to high larvae survival, but we attempted to estimate the safety of the desired *in vivo* dose: considering that (i) the 500 mg/mL concentration corresponds to a formulation dilution of 1:2 (w/w), (ii) the volume administered in the larvae is 10 μL, and that (iii) each larvae weighs approximately 200 mg, the estimated nanoemulsion LD_50_ would be higher than 25 mg/g of the insect. If we now consider that our goal is to administer approximately 20 mg of the formulation per duct, and have six nipples injected in each rat (which weighs at least 250 g), we estimate that a dose of 0.48 mg/g of the animals would be administered, which should be safe.

Encapsulation of C6 ceramide, even in tributyrin-free nanoemulsions, increased its cytotoxicity against MCF-7 cells compared to the drug solution. Possible reasons for this effect include the more efficient nanocarrier-based delivery and its ability to avoid ceramide precipitation in the culture medium (Stover et al., 2005; Fu et al., 2015; Pepe et al., 2016). Liposomes and anionic nanoemulsions have also been demonstrated to improve ceramide cytotoxicity (Stover & Kester, 2003; Stover et al., 2005; Carvalho et al., 2017 b), but to our knowledge, this is the first study demonstrating synergism between ceramide and a nanocarrier component (tributyrin), which highlights the importance of a careful design to optimize nanocarrier properties. Another interesting feature of the ceramide-loaded nanocarrier was the lower cytotoxicity against normal cells, evidenced by a 5-fold higher EC_50_. Previous studies have suggested that ceramide delivery is less toxic to cells with an intact sphingosine/ceramide pathway, in which ceramide generation is not disrupted, and our results demonstrated that this effect was not reversed by nanoencapsulation (Selzner et al., 2001; Lopez-Marure et al., 2002; Struckhoff et al., 2004).

Intraductal administration of the optimized nanocarrier promoted mammary tissue targeting and prolonged tissue localization of C6 ceramide in comparison to its i.p. injection and to the intraductal administration of a solution. However, it should be noted that even as a solution, C6 ceramide was retained for longer periods of time (over 48 h) in the mammary tissue compared to the previously investigated fluorescein disodium, which displayed *t*_1/2_ of ∼15 min (Singh et al., 2012). This difference might relate to drug lipophilicity; with a log P >6, C6 ceramide is likely to be retained in the mammary tissue fat pad, especially after administration as a solution, which exerts no control over release (Nybond et al., 2005; Pepe et al., 2012; Cichewicz et al., 2013). Thus, the contribution of the nanocarrier developed here might be more pronounced for retention of less lipophilic drugs.

## 5. Conclusions

In conclusion, the results obtained here support the advantage of the bioadhesive nanoemulsion for localization of C6 ceramide in the mammary tissue. Considering the higher cytotoxicity of the nanoemulsion against cancer cells, and low irritation potential of the unloaded nanocarrier, it may also find applicability for local delivery of active agents useful for prevention of breast cancer in high-risk women. Finally, the selection of formulation components and the choice of drug and other active agents are important factors for nanocarrier properties due to the possibility of synergism, which optimizes the therapeutic potential without the need to increase the dose of individual agents.

## Supplementary Material

Amanda_et_al._Supplementary_Material.docx
