# Supplementary material for: Multifunctional nanoemulsions for intraductal delivery as a new platform for local treatment of breast cancer
Source: Drug Deliv. 2018 Mar 1;25(1):654–67. doi: 10.1080/10717544.2018.1440665 (PMC7011997; doi:10.1080/10717544.2018.1440665)
Supplement: Amanda_et_al._Supplementary_Material.docx [file IDRD_A_1440665_SM0917.docx]

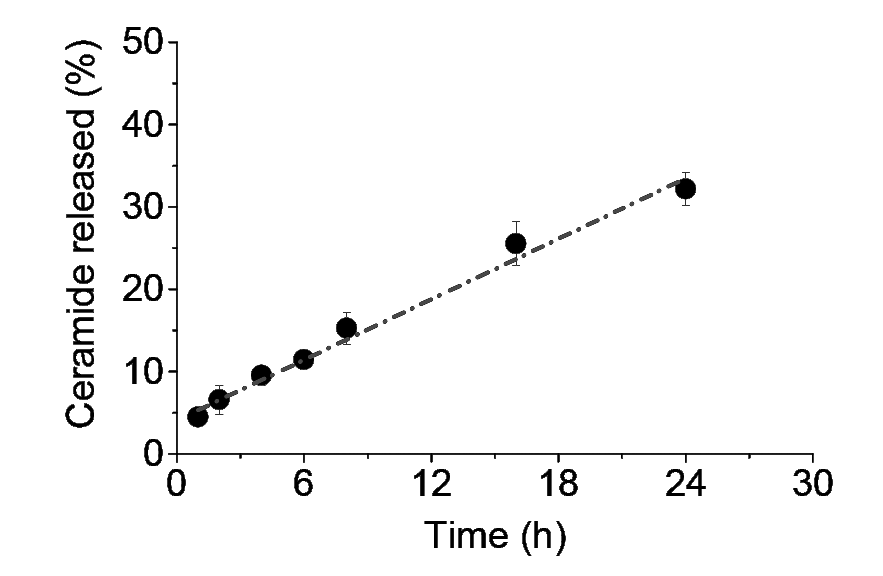


Supplementary Figure 1. Cumulative ceramide release as a function of time. Data shown as average ± standard deviation, n= 4-5.
